# 肿瘤相关中性粒细胞与肺癌的相关研究进展

**DOI:** 10.3779/j.issn.1009-3419.2019.11.07

**Published:** 2019-11-20

**Authors:** 金花 周, 树龙 蒋, 伟 王, 瑞娟 刘

**Affiliations:** 1 272000 济宁，济宁市第一人民医院 Jining No.1 People' s Hospital, Jining 272000, China; 2 250033 济南，山东大学第二医院 The Second Hospital of Shandong University, Jinan 250033, China

**Keywords:** 肿瘤相关中性粒细胞, 肺肿瘤, 肿瘤微环境, 预后, Tumor-associated neutrophils, Lung neoplasms, Tumor microenvironment, Prognosis

## Abstract

肺癌为全球性高发病率、高死亡率疾病，严重影响人类健康。其中肺部慢性炎症的长期存在与肺癌的发生发展关系密切，中性粒细胞不但参与急、慢性炎症反应，而且参与肿瘤微环境（tumor-microenvironment, TME）中的组成，与肿瘤的发生、发展密切相关。近年来研究发现，肿瘤相关中性粒细胞（tumor-associated neutrophils, TANs）在肺癌的发生和发展中发挥着重要的作用。本文就肿瘤相关中性粒细胞在肺癌中的作用、机制以及临床意义进行综述。

## Abstract (en)

Lung cancer is a global disease with high morbidity and mortality, which seriously affects human health. The long-term existence of chronic pulmonary inflammation is closely related to the occurrence and development of lung cancer, neutrophils are not only involved in acute and chronic inflammation, but also in the composition of tumor microenvironment (TME), which is closely related to the occurrence and development of tumors. Recent studies have found that tumor-associated neutrophils (TANs) play an important role in the occurrence and development of lung cancer. This article reviews the role, mechanism and clinical significance of tumor-related neutrophils in lung cancer.

## 1 前言

众所周知，肿瘤微环境（tumor-microenvironment, TME）在肺癌的发生、发展及预后中起到十分重要的作用，而慢性炎性反应是肿瘤微环境的特点之一，因此TME中的炎症细胞和介质可能在肺癌的发展中起关键作用^[1]^。1863年，Virchow检测到肿瘤组织的白细胞浸润，并提出炎症与癌症的关系，这种与癌症相关炎症的重要性逐渐被认识到，被认为是癌症的第七个特征性标志^[2]^。机体在慢性炎症免疫应答过程中，肿瘤细胞、免疫细胞和炎症三者之间错综复杂的相互作用，逐渐产生有利于血管生成和免疫抑制的TME，最终使肿瘤细胞逃避免疫系统的控制，发生癌症。肿瘤相关巨噬细胞（tumor-associated macrophages, TAMs）和中性粒细胞（tumor-associated neutrophils, TANs）是TME的重要组成部分，对几乎所有实体肿瘤的生长和进展均有影响。目前，肿瘤浸润中性粒细胞的作用尚存在争议，我们对中性粒细胞在肺癌患者中的功能的认识仍然是有限的。在本综述中，我们主要讨论中性粒细胞在肺癌发生和发展中的作用，以及它们作为肺癌临床生物标志物和治疗靶点的潜力。

## 2 肿瘤相关中性粒细胞

中性粒细胞是炎症过程的重要成分，其被视为抵抗感染的第一道防线，占人体循环中所有白细胞的50%-70%。TANs是肿瘤内浸润的中性粒细胞，在机体内肿瘤发生后，会产生相应的趋化因子，血液循环中的中性粒细胞则可被趋化因子吸引，透过血管壁进入肿瘤组织，从而形成TANs^[3]^。TANs在肿瘤从恶性转化到肿瘤进展、细胞外基质（extracellular matrix, ECM）修饰、血管生成、细胞迁移和免疫抑制等多个方面都有重要的作用^[4, 5]^。TANs具有较高的功能可塑性，其在肿瘤中既可以抗癌，也可以促癌，研究^[6]^表明TANs类似于TAMs的两面性表型，既有抑制肿瘤生长的“N1”型，又有促进肿瘤生长和恶性转移的“N2”型。直到2015年，研究人员才认识到，与癌症相关的中性粒细胞能够保持某些功能可塑性，当暴露于肿瘤微环境中时，可以进行“交替激活”^[7, 8]^，例如，转化生长因子-β（transforming growth factor-β, TGF-β）的存在可以促进肿瘤原表型（N2-TANs），而干扰素-β（interferon-β, IFN-β）的存在或抑制TGF-β信号则会导致TANs产生抗肿瘤（或N1）表型。

## 3 TANs的促肺癌作用及相关机制

TANs可通过合成、分泌相关蛋白酶参与肿瘤增殖、侵袭及转移密切相关。其中中粒细胞弹性蛋白酶（neutrophilelastase, NE）是诸多蛋白酶中最重要的一种，是防御细菌感染的重要酶类。Houghto等^[9]^最新研究表明，NE在肺腺癌的Lox-Stop-Stop（LSL）-K-ras的小鼠模型中具有促进肺癌细胞生长的作用。NE的这种作用并不是通过降解细胞外基质，而是NE对肿瘤细胞生长的直接促进作用，中性粒细胞弹性蛋白酶通过进入肿瘤细胞内直接诱导人和小鼠肺腺癌的肿瘤细胞增殖。NE不仅与肺癌的发生有关，与其远处转移也相关，Wislez等^[10]^研究了中性粒细胞在支气管肺泡癌转移中的作用，发现人肺腺癌细胞系与中性粒细胞相互作用，中性粒细胞可促使癌细胞脱落，其中涉及NE，提示NE参与肿瘤的转移。除了NE，基质金属蛋白酶（matrix metalloproteinases, MMPs）是中性粒细胞释放另一种重要蛋白水解酶，具有降解细胞外基质的功能，与肿瘤密切相关。TANs可能是MMP-9的重要来源，MMP-9是在肿瘤中参与肿瘤血管调控开关，可以促进肿瘤新生血管形成和肿瘤转移，在肿瘤发生、发展中起重要作用^[11]^。除了MMP-9，MMP-2也被发现与多种恶性肿瘤的浸润、转移有关^[12]^。而史林等^[13]^研究中通过另一面证实了MMP-9、MMP-2与肿瘤的作用，中性粒细胞可以在肿瘤微环境和抑制内源性IFN-β的条件下，上调MMP-9和MMP-2的分泌和酶活性，发挥促进恶性肿瘤发展的N2型作用。TANs除了分泌蛋白酶发挥作用外，还可以产生大量的促炎因子，如MCP-1、IL-8、MIP-1α和IL-6，以及抗炎IL-1R拮抗剂，同时还分泌其他细胞因子包括：IL-1β、TNF-α、IL-12、G-CSF、VEGF、CC家族的趋化因子，以及CXC家族的趋化因子等^[14]^。目前的研究中，在腺癌A549细胞中加入中性粒细胞可促进其增殖，而这种增殖过程中伴随着弹性蛋白酶和COX-2衍生的PGE2的释放，而抑制这些介质则可抑制肿瘤细胞的增殖。此外，吲哚美辛或特异性COX-2抑制剂NS-398对COX-2活性的干扰也使中性粒细胞存在时A549的增殖减弱。同时，在A549中性粒细胞共培养中检测到大量的COX-2依赖的前列腺素E2合成。这些发现表明中性粒细胞与肿瘤细胞间的直接相互作用导致炎症介质的释放，而炎症介质的释放又可能促进非小细胞肺癌（non-small cell lung cancer, NSCLC）的肿瘤生长。趋化因子受体CXCR2及其配体CXCL1、CXCL2和CXCL5在肿瘤相关中性粒细胞归巢到发育中起重要作用。我们发现中性粒细胞的募集受内源性IFN-β的影响，这种影响是通过调节这些趋化因子及其受体来实现的^[15]^。众所周知，TANs可以通过NE、MMP-9等促进实体肿瘤中血管的发育，从而促进肿瘤的生长和转移，研究^[16]^发现脾切除术可以减少原发性肿瘤中浸润性TAM、TAN的数量，可减少肺转移和原发性肿瘤的免疫微环境，还会影响转移前和转移部位的免疫微环境。

## 4 TANs的抑制肺癌作用及相关机制

与上述促肿瘤效应相反，几项涉及肿瘤的动物实验中发现中性粒细胞的抗肿瘤及抗转移作用。TANs抑制肿瘤细胞增殖最主要的途径是通过抗体依赖的细胞介导的细胞毒作用（antibody dependent cell mediated cytotoxicity, ADCC）减少肿瘤增殖，抗体可以通过TANs表面Fc受体来识别肿瘤，从而释放细胞毒介质，产生细胞毒作用，杀伤肿瘤细胞，TANs还可以直接产生细胞毒作用介质如ROS、髓过氧化物酶（myeloperoxidase, MPO）等，它们可以杀伤肿瘤细胞，从而抑制肿瘤细胞增殖。还有研究^[17, 18]^显示，TANs可以释放TNF相关的凋亡诱导配体（TNF related apoptosis inducing ligand, TRAIL），诱导肿瘤细胞凋亡。有研究^[19, 20]^已证实IFN-β可抑制肿瘤浸润中性粒细胞过程中血管生成因子如VEGF和MMP-9的表达，可导致IFN-β缺陷动物的肿瘤血管化和生长增强。研究^[21]^发现，在LLC肺癌模型中，Ⅰ型干扰素信号受损IFfnar1（-/-）小鼠的肺转移率高于对照组。转移前生态位的形成，以及中性粒细胞对肿瘤细胞的毒性降低，使IFfnar1（-/-）小鼠的转移过程增强。此外，IFN-β在调节中性粒细胞的生成和它们在原发肿瘤中的寿命方面起着重要的作用^[22, 23]^。肿瘤微环境中嗜中性粒细胞在实体瘤功能中的抗肿瘤作用随肿瘤类型、肿瘤进展阶段和不同治疗而异，虽然大量临床资料提示中性粒细胞浸润提示实体瘤预后不良，然而某些肿瘤疗法需要有功能的中性粒细胞才能有效，因此，调节中性粒细胞表型是一种有效的治疗选择，但介导中性粒细胞极化的因素仍未完全明确。

## 5 TANs调节其他免疫细胞对肺癌的影响

TANs除了直接作用于肿瘤细胞，还可以通过调节多种免疫细胞发挥其对肿瘤的促进或抑制作用。其发挥调节方式主要有：①招募免疫细胞。中性粒细胞分泌包括TNFR、组织蛋白酶G和中性粒细胞的细胞因子，对T细胞有直接作用并促进弹性蛋白酶增殖和细胞因子产生，招募和激活T细胞，增强整体适应性免疫抗肿瘤反应^[24]^。在早期NSCLC的研究中发现，TANs的募集可以增强CTL的免疫应答，从而抑制肿瘤发展。Eruslanov等^[25]^研究认为，TANs与活化的T细胞之间的相互作用导致中性粒细胞表面上CD54、CD86、OX40L和4-1BBL共刺激分子的显著上调，这在正反馈环中支持T细胞增殖，研究结果表明，在肺癌的早期阶段，TAN不是免疫抑制剂，而是刺激T细胞反应。先前已经表明，粒细胞髓样调节细胞，即TAN和粒细胞髓样抑制细胞（G-MDSC）能够抑制CD8 T细胞增殖并影响其活化。Michaeli等^[26]^进一步研究证明了TANs抑制了CD8 T细胞的抗肿瘤作用，并消除了它们延缓肿瘤生长的能力，其主要机制是通过TANs中的TNFα途径诱导细胞凋亡。②中性粒细胞胞外陷阱（网状结构）。网状结构是在细胞外病原体作用下从中性粒细胞释放出来的，通常由纤维去致密染色质和多聚体化组蛋白、MPO和各种细胞质蛋白组成，考虑其可能作用于原发性肿瘤内，以促进疾病的进展和传播，但具体作用及机制需进一步研究阐明，迄今为止，大多数研究只在循环的中性粒细胞中描述了这一现象^[27]^。近年来的研究^[28]^发现，在胃癌中，GM-CSF可以诱导TANs的活化，在这一过程中通过激活JAK/STAT3通路诱导PD-L1的表达，TANs以PD1/PD-L1依赖的方式抑制T细胞的活化，从而促进胃癌的进展。在肺癌中，TNAs是否也通过PD1/PD-L1影响T细胞活性，目前尚无研究。

## 6 TANs对肺癌治疗及预后的影响

慢性炎症不仅与肺癌发病、进展有关，而且影响患者的治疗效果及预后，影响往往是负面的。在目前最新的免疫治疗中，炎症反应可能会干扰免疫治疗的效果，对接受免疫治疗后的NSCLC患者，检测到的LDH、CRP、NLR、PLR和中性粒细胞、淋巴细胞和嗜酸性粒细胞计数都与总生存期（overall survival, OS）有关，低中性粒细胞绝对值、高淋巴细胞计数和高嗜酸性粒细胞计数与nivolumab治疗效果呈正相关，治疗前升高的NLR和PLR与OS和无进展生存期（progressive-free survival, PFS）呈负相关，并且与nivolumab治疗的转移性NSCLC患者的反应差相关。有时还与PFS有关，低NLR和PLR与较长的PFS相关^[29-31]^。在手术管理的NSCLC中，白细胞计数升高与可切除疾病患者的生存率相关，在一项涉及早期（Ⅰ期-Ⅲ期）NSCLC患者的研究^[32]^中，发现高CD66b中性粒细胞密度对OS的影响很小，但与手术切除后复发的发生率有关。318例患者（50%）肿瘤内CD66b阳性中性粒细胞升高，CD66b阳性细胞的增加与高累积复发率（cumulative incidence of relapse, CIR）相关（中位CIR，低CD66b阳性细胞密度为51个月；高CD66b阳性细胞密度为36个月）并趋向于更差的OS（中位OS，低CD66b阳性细胞密度为57个月；高CD66b阳性细胞密度为54个月）。Rakaee等^[33]^描述了TANs在早期NSCLC患者中的相对亚型特异性预后意义：CD66b TANS在鳞状细胞癌患者中的存在被描述为一个积极的预后因素，而在腺癌中则被描述为一个不利的预后因素。相反，在另一项研究^[34]^中，高中性粒细胞计数与鳞癌患者的预后不良有关，而与腺癌预后无关。

## 7 总结及展望

中性粒细胞在肺癌的发生和发展中有着重要的贡献，近年来逐渐被关注。但目前大多数关于中性粒细胞在肺癌中作用的研究都是基于动物实验，临床资料大都是从外周血中性粒细胞的分离中获得的。肺癌患者TANs表型和功能的数据仍然有限，而且大多来自早期疾病患者。本综述重点阐述了TANs在肺癌中的作用及相关机制，包括对肿瘤细胞的直接和间接作用，以及对肿瘤微环境及其免疫细胞含量的间接影响。通过对中性粒细胞表型及功能的改变，临床上可能成为TANs在癌症中作用研究的重要结果，并可能促进新一代免疫疗法的发展。
